# Longitudinal stability of the diurnal rhythm of intraocular pressure in subjects with healthy eyes, ocular hypertension and pigment dispersion syndrome

**DOI:** 10.1186/1471-2415-14-122

**Published:** 2014-10-15

**Authors:** Cord Huchzermeyer, Udo Reulbach, Folkert Horn, Robert Lämmer, Christian Y Mardin, Anselm GM Jünemann

**Affiliations:** Department of Ophthalmology, University Hospital Erlangen, Friedrich-Alexander-University Erlangen-Nuremberg, Schwabachanlage 6, 91054 Erlangen, Germany; Department of Public Health and Primary Care, Trinity College, Dublin, Ireland

**Keywords:** Glaucoma, Intraocular pressure, Diurnal rhythm, Ocular hypertension, Pigment dispersion syndrome

## Abstract

**Background:**

The diurnal fluctuation of intraocular pressure may be relevant in glaucoma. The aim of this study was to find out whether the timing of diurnal fluctuation is stable over the years.

**Methods:**

Long-term IOP data from the Erlangen Glaucoma Registry, consisting of several annual extended diurnal IOP profiles for each patient, was retrospectively analyzed. Normal subjects, patients with ocular hypertension and with pigment dispersion syndrome were included because these subjects had not been treated with antiglaucomatous medications at the time of data acquisition. A cosine curve was fitted to the IOP data and the stability of individual rhythms over the years was tested using the Rayleigh test. To compare the peak times among groups, means were calculated only from subjects with a significant Rayleigh test.

**Results:**

Of the fifty-two eligible subjects, a total of 364 extended diurnal IOP profiles measured in a sitting position had been collected over a period of 114 ± 39 months. The Rayleigh test indicated intraindividual stability of phase timing only in 19 subjects (36%). In subjects with pigment dispersions syndrome, peak IOP occurred on average two hours and seven minutes later during the day compared with subjects without this condition (p = 0.05).

**Conclusions:**

Fitting of cosine curves to the clinical IOP profiles was generally feasible, although careful interpretation is warranted due to lack of measurements in supine position and between midnight and 7 am. The interesting observation of a phase lag in eyes with pigment dispersion syndrome warrants confirmation and exploration in future prospective studies. The analysis of the IOP data showed no stable individual rhythm in the long term in a majority of patients.

## Background

Open-angle glaucoma is a chronic neurodegenerative disease with progressive loss of retinal ganglion cells and optic nerve fibers accompanied by characteristic cupping of the optic nerve head. In clinical practice, intraocular pressure (IOP) is the most important risk factor for glaucoma because lowering of IOP is the only therapeutic option in glaucoma whose effectiveness has been proven in large randomized clinical trials [1–3].

IOP fluctuates rhythmically with a 24-hour period (diurnal variation), but it also shows rhythmical fluctuations with other phase lengths and sporadic fluctuation [4]. Diurnal variation of IOP has been examined in clinical studies for a long time, because it was felt that “it concerns itself with one of the most fundamental aspects of the disease” [5]. In eyes with glaucoma, timing of IOP variation was believed to be generally more erratic [6]. Newer studies support this notion, and it has been demonstrated that the responsible circadian timing system can be altered in glaucoma [7]. Furthermore, a larger magnitude of IOP fluctuations is generally considered characteristic of glaucoma [5].

In the beginning, curves have been simply classified into a morning type, a night type etc. according to the time of day that the IOP peak occurred [6, 8]. In newer cross-sectional studies [8–12], phase timing of IOP curves was studied using cosinor analysis: this mathematical technique uses a cosine curve with a 24-hour period as a model of IOP fluctuation [13]. In these carefully designed studies, that were carried out in a sleep lab, a physiological rhythm was found in normal eyes with an IOP that is highest at night [9, 10]. This rhythm was synchronized between subjects when measurements were corrected for individual differences in the sleep-wake-cycle, and a phase lag was observed in aging subjects and in eyes with early glaucoma [8, 14].

To our knowledge, cosinor analysis has never been used to analyze diurnal rhythms of IOP in studies with a long-term follow-up. Duke-Elder has hypothesized that “each individual has a characteristic rhythm which is obstinately maintained” [5]. A test of this hypothesis and the characterization of longitudinal changes in glaucoma are desirable. The gold-standard for such a study would clearly be a prospective trial in a sleep lab with control over many aspects of the environment. However, such studies need a lot of resources, and it is difficult to acquire funding because, ultimately, the clinical importance of diurnal rhythm of IOP in glaucoma has not yet been proven.

Therefore, it would be helpful, if research on IOP rhythms could be performed retrospectively on existing IOP data, despite the limitations that are always associated with such a study design. This may allow to form better hypotheses and to clarify of the importance of IOP rhythms in glaucoma before conducting a prospective trial.

The aim of this retrospective analysis was to find out whether cosinor analysis can be used on clinical IOP profiles and whether individual IOP rhythms are reproducible in the long-term. Clinical extended diurnal IOP profiles with careful data acquisition were collected over many years on a number of patients for the Erlangen Glaucoma Registry – an ongoing prospective observational study focused on morphological and functional diagnostic methods in glaucoma. In this first analysis, only subjects without manifest glaucoma (normal subjects and patients with ocular hypertension or pigment dispersion syndrome) were included because they were not under antiglaucomatous treatment at the time of the measurements.

## Methods

This study is a secondary, retrospective analysis of longitudinal IOP data from an ongoing prospective, observational study.

### The Erlangen Glaucoma Registry

The primary study, the Erlangen Glaucoma Registry (EGR; ClinicalTrials.gov number NCT00494923), is concerned with subjects at risk for glaucoma and patients with manifest glaucoma, its special focus being the predictive value of diagnostic tests. It is a local registry, located at the University Eye Hospital Erlangen. The study was approved by the ethics committee of the Friedrich-Alexander-University Erlangen-Nuremberg and conducted in accordance with the tenets of the declaration of Helsinki. The first examinations took place in 1991. The EGR has been supported by the Deutsche Forschungsgemeinschaft (DFG) until July 2009, first by the DFG Clinical Research Group and then, since 1997, by the SFB 539 grant. Subjects who agreed to undergo extensive diagnostic testing at regular follow-up visits were recruited from the glaucoma clinic of the University Eye Hospital Erlangen. All subjects gave written informed consent.

Complete clinical examinations were performed annually; they included slit-lamp examination, standardized perimetry, dilated fundus examination, standardized photography of the optic nerve head, and a 24-hour IOP profile (see below for details). Intermittent examinations were scheduled as needed. When indicated, patients were treated conservatively or surgically according to the current clinical standards. Careful slit-lamp examination of the anterior segment was performed to detect PDS and to look for signs of other ocular conditions, especially ones that may cause secondary glaucoma, like pseudoexfoliation syndrome. Standardized automatic white-on-white perimetry was performed with an Octopus 500 perimeter (Haag-Streit, Switzerland) using the G1 program. When patients were not experienced in sensory tests, the first three visual fields were not included in the analysis. Standardized 15°-fundus photographs centered on the optic disc were performed annually. They were graded in a masked fashion by two glaucoma specialists for signs of glaucomatous atrophy. Each year, the complete series of photographs was analyzed for loss of neuroretinal rim tissue or progressive optic disc cupping by the specialists.

These data were entered into a database and checked for internal consistency on a regular basis.

### Selection of eligible patients and study groups

For this retrospective analysis, data from the EGR was used with permission of the principal investigators and the medical director of the University Eye Hospital Erlangen. Only subjects without signs of manifest glaucoma (i.e. normal visual field and normal appearing optic nerve) were included and only if regular follow-up of more than four years had been documented and at least four complete IOP profiles had been performed without local or systemic antiglaucomatous therapy. Patients with manifest glaucoma or with other eye disease (except cataract) were excluded. Likewise, shift workers were not included in the study. Eligible subjects from the Erlangen Glaucoma Registry with appropriate diagnoses and length of follow-up were identified by querying the database. Inclusion and exclusion criteria were then verified by the authors using data from the database or from the clinical records of these participants. None of the subjects included in this retrospective analysis had signs of glaucomatous optic disc changes or visual field defects. Subjects with pseudoexfoliation, rubeosis iridis, angle closure, aphakia, uveitic or congenital glaucoma were excluded. Furthermore, no subject had undergone intraocular surgery, except cataract extraction.

The selected subjects were divided into the following three groups: 1) normal subjects with normal intraocular pressure and without signs of pigment dispersion (normals); 2) subjects with ocular hypertension who showed no signs of pigment dispersion, but three intraocular pressure readings ≥ 23 mmHg (OHT); and 3) subjects with at least two signs of pigment dispersion syndrome, regardless of IOP (PDS). Clinical signs of PDS include melanin pigment in the corneal endothelium (Krukenberg spindle), melanin pigment on the iris and the lens, or typical radial transillumination defects of the iris.

The subjects that were included in the “normal” group had been referred to us because they were classified as glaucoma suspects elsewhere. Commonly, the reason was an elevated IOP measured by the referring ophthalmologist before inclusion in the EGR. Many of these subjects had a positive family history for glaucoma. These subjects were concerned and chose to continue participating in the study despite being informed that there were no signs of either OHT or glaucoma after the first few visits.

### Intraocular pressure profiles

IOP measurements, including those at night, were performed in a sitting position using Goldmann applanation tonometry. Diurnal intraocular pressure profiles were performed annually and consisted of a series of at least six measurements. Physical activity, exact timing of the sleep-wake-cycle, and consumption of tobacco, caffeine or alcoholic beverages were not recorded. In general, subjects were instructed not to stay up and wait for the midnight recording, but rather go to bed at their usual time. The subjects underwent further morphological and sensory tests during the first half of the day between IOP measurements, but all tests requiring dilatation of the pupil were performed on the second day after the last IOP measurement.

Most subjects were hospitalized for one day and one night for regular measurements at 08:00 h, 12:00 h, 17:00 h, 21:00 h, and 24:00 h. Subjects were accommodated in a hospital room, usually with one or two other patients. For the measurement at midnight, patients were woken by a nurse not more than 15 minutes before IOP was measured in the office a few meters from the patient’s room. The next day, an additional measurement was performed at 07:00 h immediately after the subject was woken.

### Cosinor analysis

Clinical data, including IOP measurements, were entered into an Oracle database; the database was checked for internal inconsistencies on a regular basis. For statistical analysis, IOP data were exported from the database into a text file (comma separated values) and analyzed using the R statistical software (R: A language and environment for statistical computing, R Foundation for Statistical Computing, Vienna, Austria). For each patient, one eye was selected for statistical analysis. Cosine curves with a period length of 24 hours based on the following equation were fitted to the IOP profiles with the least-squares method using the function nls provided by the package stats of R.
1

**Figure 1:**
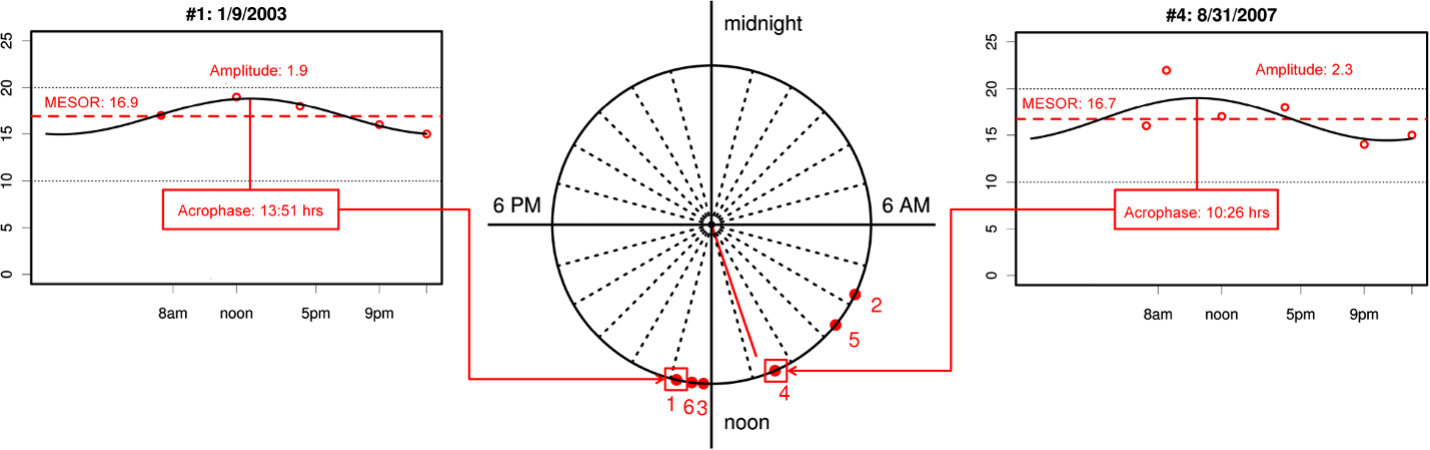
**Cosinor analysis of IOP data of one subject.** For this subject, six annual 24-h IOP profiles, each consisting of at least 6 measurements, were available for analysis. A cosine curve was fitted to each series. The cosine curve fitted to the first 24-h series, shown on the left side, fits very well, whereas the cosine curve for series number 4, on the right side, exhibits only a mediocre fit. The mean IOP level estimated by the cosine model, called MESOR, is similar in both cases (between 16 and 17 mmHg). The middle of the figure shows a clock plot with the time of day at which the curves fitted to each 24-h series peaked (i.e. the acrophase). All acrophases are plotted on the unit circle. Although it may be interesting in certain situations to use the distance to the center to symbolize the amplitudes of the cosine curves, it is not useful in this case, because it prevents the calculation of the overall direction of acrophases (i.e. mean acrophase). Circular data warrant special mathematical analysis: to calculate the overall direction of acrophases, the X- and Y- coordinates of the points shown in the clock plot are averaged separately. The vector formed by connecting the origin and the point defined by the mean X- and mean Y-coordinate indicates not only the mean direction of acrophases but also the dispersion of acrophases around the circle. A long vector signifies a small dispersion or a high stability in phase timing. The Rayleigh test is used to test the significance of the overall distribution, being significant when it is highly unlikely that acrophases are equally likely any time of day (i.e. uniformly distributed).

**Figure 2:**
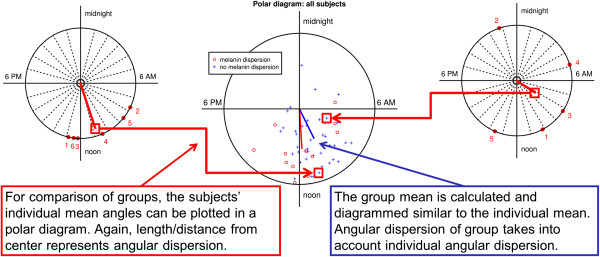
**Comparison of acrophase data between groups of subjects.** This diagram shows acrophase distribution of one subject on the left and another subject on the right side. For each subjects a mean vector, representing the long-term mean acrophase was calculated (see Figure 1). The clock plot in the middle shows the end points of these mean vectors for all subjects; vectors from the two subjects are highlighted by a red box. Two groups are shown: subjects with pigment dispersion are represented by red circles, the ones without by blue crosses. Again the analysis of time series and other circular data requires special methods, so for second-order analysis (i.e. comparing group means from already aggregated individual data), the group mean was calculated similarly to the individual mean by averaging the x- and y-coordinates. The significance of the differences of the mean angles among groups was tested using a parametric test according to Batschelet and Hotelling.

Cosine curves were characterized by three parameters: MESOR M, amplitude A, and acrophase φ. The MESOR is the **M**idline **E**stimating **S**tatistic **O**f **R**hythm, i.e. the value about which fluctuation occurs. The acrophase is the time of the highest point of the fitted curve. In Equation 1, φ is a negative value (unit: radians) that represents the phase angle of the lag behind the reference time (=midnight); however, in this paper, acrophase data are expressed as the time of day when the peak of the cosine curve occured (i.e. hours after midnight).Circular data, such as the calculated acrophases, had to be evaluated using specialized statistical methods: mean acrophases were calculated in two steps. First, X- and Y-coordinates of the points where the lines through the origin whose direction represented the acrophases in the clock plot intersected the unit circle were calculated. In a second step, all X- and Y-coordinates were averaged separately and the coordinates of the resulting point were converted back into polar coordinates. The angle between the resultant vector and the Y-axis now represented the mean acrophase, and its length r the dispersion of the angles from which it was calculated (r close to zero means high dispersion of the angles that were averaged, while r close to one signifies low dispersion). These steps are also illustrated in Figures 1 and 2. Angular standard deviation s’ was calculated from angular dispersion r according to the following equation.
2

To test for intra-individual stability of phase timing from year to year, the Rayleigh test was used (implemented in R in the Circular Statistics package by Ulric Lund and Claudio Agostinelli, 2007; circular: R package version 0.3-8). A significant test indicated the presence of a significant predominant long-term acrophase as opposed to a uniform, random distribution of acrophases around the circle.

To test for differences in mean acrophase between groups, a second-order parametric analysis of two samples of angles was performed using a method proposed by Batschelet and Hotelling (Figure 2). This test was implemented manually by the authors after the description in Zar [15].

Categorical data were analyzed using Fisher’s exact test, and comparisons of clinical parameters among groups were made using analysis of variance (ANOVA), followed by the Tukey HSD post-hoc test if the ANOVA was significant. Generally, a level of p ≤ 0.05 was considered significant.

## Results

Fifty-two subjects who met the inclusion criteria were identified from the database in the beginning of 2010. Of these 52 subjects, 30 were normal, 11 had PDS, and 11 had ocular hypertension (at least three IOP readings ≥ 23 mmHg). All subjects had normal visual fields and normal optic disc morphology at baseline (Table 1), and no subject developed glaucomatous changes during the follow-up between 50 and 202 months (mean follow-up 113.9 ± 39.1 months).

**Table 1:** **Global indices of visual field and optic disc morphology**

Group	All	Control	OHT	PDS	p ^a^
n	52	30	11	11	-
MD [dB]	0.3 ± 1.1	0.6 ± 1.1	0.0 ± 1.1	−0.2 ± 1.2	0.08
PSD	1.6 ± 0.4	1.7 ± 0.3	1.6 ± 0.5	1.6 ± 0.3	0.39
Disc area [mm^2^]	2.86 ± .80	2.93 ± 0.81	2.74 ± 0.87	2.75 ± 0.74	0.78
Vertical C/D	0.57 ± 0.17	0.61 ± 0.14	0.47 ± 0.24	0.57 ± 0.14	0.08

^a)^ANOVA.

### Cosinor analysis

Cosinor-analysis of the 364 suitable IOP profiles available for these 52 subjects was generally feasible, and only three IOP profiles (1%) had to be excluded because the algorithm of the function nls for the least-squares method in the R statistics software was not able to calculate an adequate cosine model. On qualitative examination of the remaining IOP profiles, the cosine models seemed adequate to describe fluctuation of IOP and the fit seemed well constrained by the data in the majority of cases.

### Reproducibility of cosinor analysis

The variability of the individual acrophases from visit to visit was higher than expected; circular standard deviation was between 48 minutes and as much as 7 hours 50 minutes with a median of 4 hours 2 minutes. Due to this variability, the Rayleigh test showed a statistically significant mean direction only in 19 of the 52 subjects (36%).

From the clinical files, we found out that not all subjects had been willing to follow the official protocol with IOP profiles in the hospital at all times. These subjects had left the clinic at 17:00 h, and had returned at 20:00 h, at 23:00 h, and at 08:00 h the next morning for IOP measurements (regarding the primary focus of the Erlangen Glaucoma Registry, this was considered a negligible deviation from the originally intended protocol). This had occurred either only at some (“mixed protocol”) or at all visits (“ambulatory patients”). On average, subjects who were hospitalized for all IOP profiles had similar curve parameters compared to subjects that followed the ambulatory protocol or those that were hospitalized only on some occasions. Especially, the mean acrophases were not different (Table 2). However, subjects that were always hospitalized for IOP curves were significantly more likely to have a significant Rayleigh test than ambulatory subjects or those who were hospitalized on some occasions (61.5% vs. 25.0% vs. 30.4%, respectively, p = 0.05).

**Table 2:** **The parameters of cosinor analysis for different groups of subjects**

Subjects	n	MESOR [mmHg]	Amplitude [mmHg]	Acrophase [h:min]	Circ. SD [h:min]
All	52	16.0 ± 2.3	1.9 ± 0.6	10:37	4:52
Normal	30	15.4 ± 1.5	1.7 ± 0.4	10:32	5:15
OHT	11	19.0 ± 2.0^b,c^	2.6 ± 0.7^b,c^	09:42	4:06
PDS	11	14.9 ± 1.8	2.0 ± 0.6	11:48	4:21
Young^a^	26	16.2 ± 2.1	1.9 ± 0.5	10:56	4:30
Old^a^	26	15.9 ± 2.4	1.9 ± 0.7	10:14	5:12
Hospital	13	15.7 ± 1.8	2.1 ± 0.6	10:51	3:42
Ambulatory	16	16.6 ± 2.5	2.0 ± 0.7	10:22	4:53
Mixed	23	15.8 ± 2.3	1.7 ± 0.6	10:36	5:34

MESOR: Midline Estimating Statistic Of Rhythm, i.e. mean IOP level of the cosine curve; acrophase: time of peak of cosine curve; circ. SD: circular standard deviation.

^a)^Young: ≤ median age of 44.4 years; old: > 44.4 years.

^b)^Tukey HSD test significant vs. normal (only performed if ANOVA was significant).

^c)^Tukey HSD test significant vs. PDS.

Age did not seem to influence either stability or timing of IOP rhythm. When subjects younger than the median age of 44.4 years were compared to older subjects, no statistically significant differences were found. This did not change when only subjects with significant Rayleigh tests were included, or when the lowest age quartile was compared to highest (data not shown).

### Group statistics from averaged longitudinal data

The overall directions of individual long-term mean acrophase vectors showed a higher inter-individual variability than expected but were not random: Rayleigh test demonstrated, that its distribution was significantly different from a uniform distribution (p = 0.01). Individual mean acrophases ranged between 0:13 h and 18:44 h, most subjects (62%) having a mean acrophase between 06:00 h and 12:00 h. Sixteen subjects (31%) had an acrophase later than noon, and four subjects (8%) had an acrophase earlier than 06:00 h. However, in the PDS group, the majority of subjects had a mean acrophase later than 12:00 h (54% compared to 20% in other subjects, p = 0.05).

**Figure 3:**
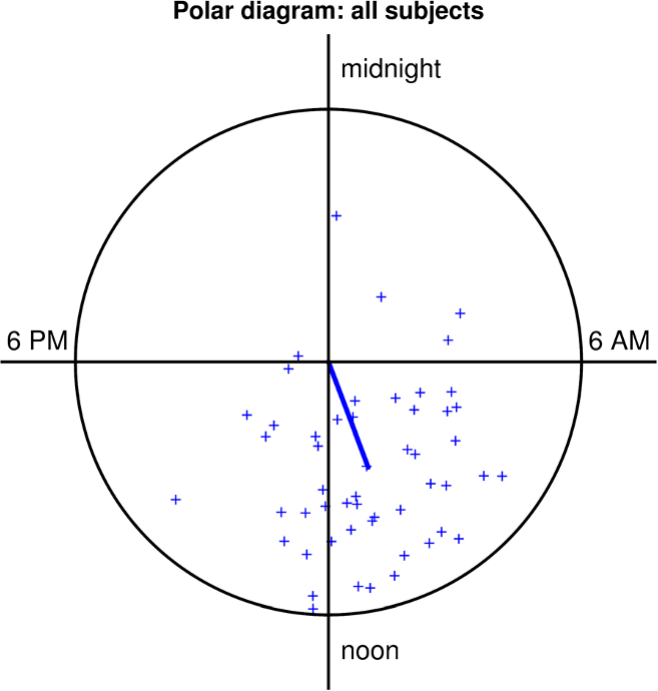
**Mean acrophase of all subjects.** Polar diagram of all 52 subjects included in the study. The majority showed a mean acrophase between 06:00 h and 12:00 h.

A detailed description of the estimated parameters of the fitted cosine curves of all subjects is presented in Table 2, and individual mean acrophases are displayed in a clock plot for each patient in Figure 3. Briefly, the average MESOR of the fitted cosine curves was 16.0 ± 2.3 mmHg and the mean amplitude was 1.9 ± 0.6 mmHg. Mean acrophase was 10:37 h with a circular standard deviation of 4 hours and 52 minutes. For differences among groups, see Table 2.

Subjects were examined annually; therefore each subject was examined at the same time every year. But annual examinations of different subjects were distributed uniformly around the year (Rayleigh test: p = 0.09), and differences in phase timing between subjects cannot be explained by seasonal variation.

### Comparison after exclusion of subjects without reproducible IOP rhythm in the long-term

For further analysis, subjects without a significant individual Rayleigh test were excluded. Estimates of MESOR and amplitude, as well as mean acrophase, did not change significantly after exclusion: MESOR was 16.2 ± 1.8 mmHg (not significantly different from that of excluded subjects) and amplitude was 2.1 ± 0.7 mmHg (again not significantly different compared to excluded subjects). After exclusion, the mean acrophase direction was 10:55 h with a circular standard deviation of 3 hours 2 minutes.

**Figure 4:**
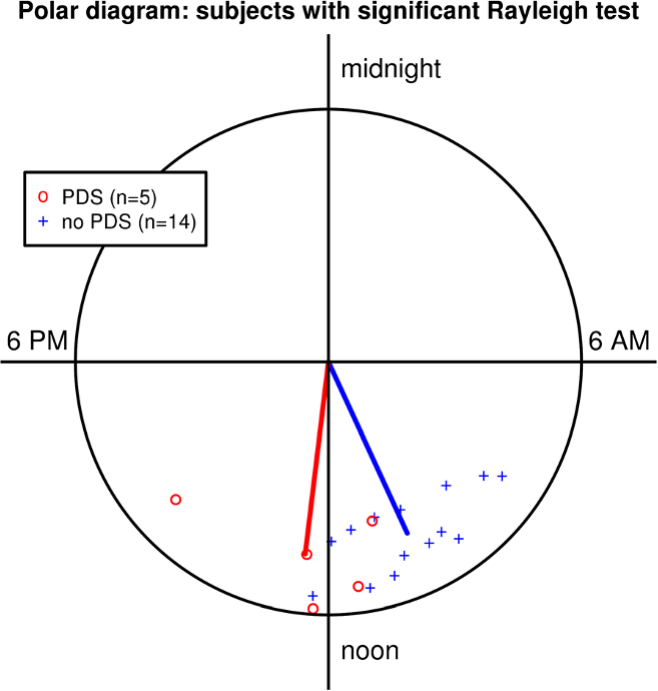
**Clock plot of the long-term mean acrophases of subjects with PDS.** Subjects with PDS (red) show a significant phase lag compared to normal subjects (blue; p < 0.05). Only the 19 subjects who had a significant Rayleigh test were included.

The individual circular standard deviation of acrophases between visits was lower in the remaining 19 subjects compared to the excluded subjects, ranging between 48 minutes and 3 hours 36 minutes, with a median of 2 hours 28 minutes. These subjects also had more similar mean acrophases (ranging between 08:13 h and 12:26 h), i.e. less inter-individual variation. Again, the distribution of mean acrophases was significantly different from a uniform distribution (p = 0.01). Fifteen subjects (79%) had an acrophase between 06:00 h and 12:00 h, and four subjects (21%) had an acrophase later than noon. Of the four subjects with an acrophase later than noon, three (75%) had PDS.Cosinor analysis of the remaining 19 subjects with significant Rayleigh test demonstrated significant differences among groups. Subjects with OHT had significantly higher MESORs and amplitudes compared to normal subjects (p = 0.001). In contrast, phase timing was not different (p = 0.17). In contrast, subjects with PDS had a significant phase lag of 2 h 7 min compared to subjects without PDS (12:28 h in the former compared to 10:21 h in the latter group, p = 0.05, Figure 4). When compared to normal subjects, the mean mesor of the PDS group was slightly lower and the amplitude was similar (p = 0.08 for MESOR, p = 0.9 for amplitude). Of the subjects with a significant Rayleigh test and PDS, 60% had an acrophase after noon compared to 7% in the subjects without PDS (p = 0.05).

## Discussion

The present study shows that the cosinor method can be used to retrospectively analyze diurnal variation in clinical data, but that it is associated with a number of limitations. In two-thirds of the subjects, the rhythm was not reproducible in the long-term.

Technically, it was possible to fit a cosine curve to the data and the fit seemed well-constrained in the majority of cases. Generally, variations of IOP can be analyzed quantitatively using sophisticated mathematical methods. For some of these methods, like autocorrelation, Fourier analysis, or Enright periodograms, time intervals between measurements have to be evenly spaced and sufficiently short [16]. Thus, we performed cosinor analysis, a method which is based on the assumption that a cosine curve is an adequate mathematical model to describe diurnal fluctuation of IOP [13]; the superimposed IOP fluctuations that do not follow a 24-hour rhythm are then considered biological noise [17]. This method has been widely used in research on IOP variation [9, 11, 18]. We have not measured IOP between midnight and 7 am. Theoretically, if a cosine curve is a good model of rhythmic IOP variation and there is not too much sporadic fluctuation superimposed, cosine analysis still yields valid results, because the peak in the early morning hours can be inferred from the other data. However, due to the lacking measurements between midnight and 7 am, the risk of an inaccurate estimate increases disproportionately high if rhythmic variation of IOP deviates from a cosine curve or if large sporadic fluctuations are superimposed.

On average, the IOP was lowest at the last measurement at midnight. Moreover, the IOP peaks occurred mostly early during the day between 6 am and noon. These findings are in line with older clinical studies [19, 20], but they differ from results from prospective studies in sleep labs [9–11]. This indicates that we may have missed IOP peaks in the early morning hours due to the gap in our clinical data between midnight and 7 am. Another reason for the lower at night in our study may have been underestimation of nocturnal IOP by measuring in the sitting position. However, this cannot be the only reason because, contrary to our data, Liu et al. [10] have found higher IOP at night despite measuring in a sitting position, whereas Quaranta and coworkers [21] have not found a systematic increase of IOP at night even when measuring in a habitual position (i.e. sitting position during the day, supine at night). Therefore, the ultimate reason for the different timing of IOP peaks in different studies is not known – it may be associated with other details of the setting and the protocol of the measurements.

In our study, the IOP rhythms were not reproducible in the long-term. Thus, the notion of an individual IOP rhythm as it was postulated by Duke-Elder [5] cannot be confirmed by our data. Currently, it is not clear how robust the rhythm of this 24-h variation is in the long-term [16], that is how much the pattern of IOP curves changes over the years. The reproducibility of IOP rhythms, even in the short term, has recently been questioned [22–24]. This is supported by our data: only in one third of our subjects, the Rayleigh test showed statistically significant mean direction of acrophases over the years. This variability in the timing of IOP variation may be explained by synchronization of the internal clock to environmental influences, by external rhythmic influences on IOP, or by the inability of cosinor analysis to detect an underlying stable 24-hour rhythm in some cases due to superimposed non-rhythmic IOP variations, like physical activity, or intake of caffeine or alcohol. Newer studies that attempt a continuous measurement of IOP in primates and humans also show that the behavior of IOP is very dynamic [22, 25].

We were able to demonstrate that IOP rhythms were more reproducible when the environment was more strictly controlled. Only 16 of the 52 subjects were hospitalized each year for acquisition of the IOP curve. These subjects were significantly more likely to have a stable rhythm of IOP in the long term than subjects in whom ambulatory IOP profiles were performed on some occasions or even every single time. In other words, the more standardized the environment, the higher the stability of acrophase over the years. On the other hand, although a highly standardized environment is desirable in a scientific setting, it may limit the external validity of the resultant IOP curves.

Therefore, the interpretation of these results is limited by the study design, and the value of cosinor analysis for retrospective examination of IOP rhythms in longitudinal clinical data may be limited. However, in subjects with stable rhythm a phase lag in subjects with PDS was observed. This is an intriguing finding, although it does not seem to have any immediate clinical applications.

The reasons for the observed phase lag in IOP rhythm in subjects with PDS are not known, and they cannot be concluded from our data. Changes in biological rhythms are generally caused by one of three conditions: altered phase timing of the endogenous clock, alterations of the effector mechanisms, or external influences [26]. One possible explication that comes to mind in PDS are rhythmic changes in the release of pigment granules into the anterior chamber [27]. Rhythmic changes in the amount of pigment released may be caused either by increased physical activity during the day [28, 29], or by changes in diameter of the pupil [30]. Although no explanation can be given from our data, it may indicate that beside hormonal and neural mechanisms, local factors may modify the diurnal variation of IOP. Because of the limitations of the retrospective study design, this finding needs to be confirmed in a carefully designed clinical study. This finding may indicate that in some instances, cosinor analysis may in fact be applied to generate hypothesis from clinical data.

The importance of diurnal fluctuation of IOP for development or progression of glaucoma is subject to controversy and cannot be discussed in much detail here; please see Sultan et al. [31] and Quaranta et al. [4] for a thorough review of the literature. Most studies are concerned with the magnitude of IOP fluctuations, either completely disregarding the timing of these fluctuations [32, 33] or distinguishing mainly between peaks during and outside office hours [34, 35]. However, very little is known about the importance of IOP rhythms for progression. More important, clinical studies that do show association between changes in IOP rhythms and glaucoma [5, 6, 14, 36] cannot distinguish between cause and effect. Only Noël and coworkers [12] have not found any differences in IOP rhythm between healthy volunteers and glaucoma patients. Experimental studies, for example in an animal model, are needed to clarify this. It seems likely that the influence of IOP rhythms on glaucoma progression cannot be analyzed without looking at the circadian variation of other biological factors, especially blood pressure [4, 30].

### Limitations

The presented study is a retrospective analysis of data that was not primarily intended for studying chronobiology of IOP. Measurements were not equally spaced throughout the 24 hours and no measurements were made between midnight and 08:00 h. Significant changes in IOP that may have occurred during this time period may have been systematically missed. Another limitation of the present study is the relatively small sample size. Thus, confirmation of the presented findings in a study with more subjects is desirable.

Furthermore, the time of sleep onset and offset was not recorded in our study, and no independent markers showing a circadian rhythm (such as body temperature or blood cortisol level) were monitored. As a result, we cannot be certain to what degree fluctuations were caused by circadian rhythms or by rhythmic environmental influences [26]. Short exposure of subjects to light may have taken place during the measurements at 21:00 h and 24:00 h. The influence of this exposure cannot be determined; however, in a study by Liu et al., short exposure to low levels of light did not seem to cause relevant disruption of the pattern of IOP fluctuation [37].

All IOP measurements, including those at midnight, were taken in a sitting position using Goldmann tonometry. It is known that posture does influence IOP, and there is a significant increase of IOP in the supine position [38]. Consequently, habitual variation of IOP (i.e. when all measurements are taken in the habitual position for that time of day) exhibits a different pattern due to the postural increase in IOP in the supine position at night [9, 10]. The lack of measurements in the supine position in our study may limit the validity of the absolute IOP measurements at night and may have lead to a different pattern of IOP variation, but it should not have influenced our primary end-point: the long-term stability of diurnal IOP curves.

In the final analysis, a bias may have been introduced by the exclusion of subjects with a non-significant Rayleigh test. On the other hand, the interpretation of long-term data from subjects with high variability in phase timing from year to year would have been difficult. Selection of any single IOP curve or comparison of accumulated individual IOP curves (that is individual mean acrophase) seemed pointless in subjects with an erratic pattern of IOP variation.

### Further research

The interesting findings of our retrospective analysis should ideally be confirmed and further explored in prospective studies. These studies might include control of the wake-sleep-cycle as well as the light–dark-cycle and control of the activity of the subjects. The latter can be achieved with a wrist device. If possible, an independent marker of the circadian system, for example, body temperature or plasma cortisol levels, should also be determined several times per day.

Specific factors associated with PDS should be examined more closely. The role of physical activity on the diurnal fluctuation of IOP in subjects with PDS may be clarified by comparing IOP profiles under regular activity to IOP profiles under bedrest conditions, similar to the study by Buguet et al. [11]. In addition, the concentration of melanin granules in the anterior chamber using a laser flare-cell meter should be determined several times during the 24-h cycle [39, 40].

In the long term, continuous measurement of IOP would be desirable. Initial experiments have been conducted using a telemetry system with an intraocular manometer in non-human primates and a contact lens in humans [22, 25]. However, methods to analyze these complex IOP curves have not yet been established.

## Conclusions

Fitting of cosine curves to the clinical IOP profiles was feasible, but the resultant curves showed no constant synchronized rhythm in the long term in a majority of patients. This may indicate that there was no stable individual rhythm, but we cannot rule out that an underlying rhythm may have been missed due to limitations of data collection. Therefore, the use of clinical data for retrospective analysis of diurnal rhythms may be limited. However, the finding of a phase lag in eyes with PDS may be carefully interpreted as a successful example of generating a hypothesis using clinical data. It warrants confirmation and exploration in future prospective studies.
